# MitraClip Procedure in Advanced Heart Failure and Severe Mitral Regurgitation: Case Report and Literature Review

**DOI:** 10.3390/jcm14031011

**Published:** 2025-02-05

**Authors:** Camilla Cirelli, Anna Merlo, Alice Calabrese, Luca Fazzini, Luigi Fiocca, Michele Senni, Massimo Iacoviello, Mauro Gori

**Affiliations:** 1 School of Medicine and Surgery, University of Milan-Bicocca, 20126 Milan, Italy; c.cirelli1@campus.unimib.it (C.C.); a.merlo@campus.unimib.it (A.M.); msenni@asst-pg23.it (M.S.); 2 Cardiology 1 Unit, ASST Papa Giovanni XXIII, 24121 Bergamo, Italy; acalabrese@asst-pg23.it (A.C.); lfiocca@asst-pg23.it (L.F.); 3 Clinical Cardiology Unit, Department of Medical Sciences and Public Health, University of Cagliari,09042 Cagliari, Italy; luca.fazzini10@gmail.com; 4 Department of Medical and Surgical Sciences, University of Foggia, 71122 Foggia, Italy; massimo.iacoviello@gmail.com

**Keywords:** MitraClip, advanced heart failure, cardiogenic shock

## Abstract

Mitral regurgitation (MR) is a common valvular disorder often seen in a severely dilated left ventricle (LV) and reduced LV function. In chronic heart failure (HF), severe functional MR increases preload, wall tension, LV workload, and worsening prognosis. The MitraClip device offers a percutaneous treatment option in HF, although its safety and efficacy in advanced and acute HF remain a gray zone. We present a successful case of the emergent MitraClip intervention in a patient with advanced HF and review the relevant literature.

## 1. Introduction

Mitral regurgitation (MR) is a prevalent valvular disorder frequently observed in dilated or ischemic cardiomyopathy with a severely dilated left ventricle (LV) and depressed LV function [1]. In patients with chronic heart failure (HF), severe functional MR leads to increased preload, elevated wall tension, and heightened LV workload, contributing to a poor prognosis [2].

In recent years, novel percutaneous treatment procedures have emerged as alternatives to cardiac surgery for addressing this valvulopathy. Specifically, transcatheter implantation of the MitraClip device (Abbott, Abbott Park, IL, USA), based on the edge-to-edge surgical technique [3], is recognized as a therapeutic option for patients with significant functional MR who are considered to have prohibitive surgical risk [4,5]. It should be considered as a Class IIa treatment option in patients with chronic HF symptomatic despite ongoing optimized therapy, according to the COAPT criteria [6].

However, while MitraClip implantation is recognized as a therapeutic option in chronic HF [1], its safety in patients with cardiogenic shock (CS) and advanced HF (AdHF) is not well-established, and the percutaneous approach for these patients remains a “grey zone”. Although some data from the literature support the use of this therapeutic option in these settings, there are no randomized controlled trials available.

We present the clinical case of a middle-aged woman affected by advanced HF and severe functional MR. The patient had contraindications for heart transplantation (HTx) or a left ventricular assist device (LVAD) and was successfully treated with an emergent MitraClip procedure, enabling weaning from inotropic therapy and subsequent discharge. Importantly, the patient is still alive at long-term follow-up. Additionally, we provide a literature review on the use of the MitraClip procedure in patients with cardiogenic shock and AdHF.

## 2. Clinical Case

A 60-year-old woman presented to the emergency department of a spoke hospital in December 2022 with a subacute myocardial infarction, complicated by cardiogenic shock. The patient had a smoking habit and a family history of ischemic heart disease. Echocardiography showed a severely depressed left ventricle ejection fraction (LVEF) and severe functional MR. She was immediately transferred to a hub center and an emergent angiography was performed, revealing three-vessel coronary artery disease, successfully managed through angioplasty. However, the patient’s hemodynamic profile remained unstable, with hypoperfusion and hypotension. An intra-aortic balloon pump (IABP) was placed in the left femoral artery. The procedure was complicated by dissection of the left femoral artery, which required thromboendarterectomy. The IABP was reinserted in the right femoral artery. Afterwards, she was referred to our hospital, a tertiary referral hospital with heart transplant and LVAD capabilities, for evaluation of advanced therapies.

On 10 January 2023, the patient was admitted to our intensive care unit (ICU). On arrival, she was treated with intravenous furosemide and IABP support, and she did not have pharmacological inotropic support. Upon initial evaluation, her extremities were cold, and there was an apical 3/6 systolic murmur, mild lung crackles at the bases, and weak pulses in the right lower limb. Initial cardiac laboratory results are presented in Table 1, indicating a notable elevation in creatinine kinase.

Echocardiography showed a dilated LV with severely reduced LVEF (20%) and moderate–severe functional MR. An urgent computed tomography angiography (CTA) was performed, revealing diffuse severe peripheral arteriopathy due to severe atherosclerosis and a small caliber of the iliac and femoral arteries and chronic cerebral vasculopathy. A Swan Ganz catheter was placed (Table 2 for hemodynamic parameters).

Considering the CTA report, the patient was discussed within the Heart Team (HT), comprising a multidisciplinary team from critical care cardiology, interventional cardiology, cardiothoracic surgery, anesthesiologist, and vascular surgery, as well as an advanced HF specialist. She was deemed to be ineligible for LVAD and heart transplant due to severe peripheral arteriopathy. Indeed, continuous flow LVAD influences shear stress on the vasculature and can further deteriorate the already severe peripheral arterial disease. The HT leaned toward initiating Adrenaline 0.05 μg/kg/min and Sodium Nitroprusside 0.16 μg/kg/min and replacing the 40 cc balloon IABP with a 34 cc balloon with smaller introducer sheath dimensions. Additionally, the inflation of the IABP was reduced to 80% to prevent persistent ischemia in the right limb, with a program of subsequent weaning from the device.

**Figure 1:**
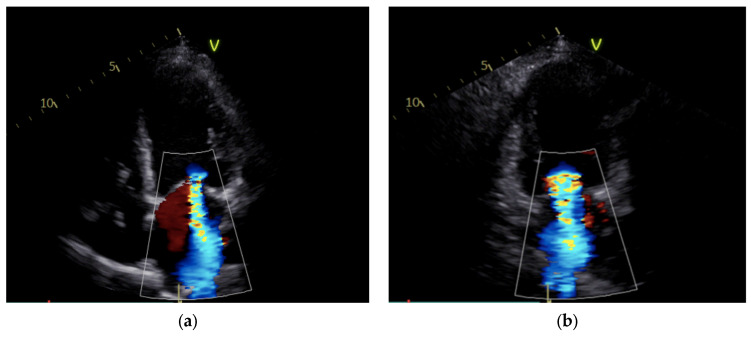
Mitral regurgitation after IABP removal: severe regurgitation is visible in apical 4-chamber view (**a**) and apical 2-chamber view (**b**).

Upon improvement of the hemodynamic condition, the IABP was surgically removed, and the patient’s low-dose inotropic support was continued. After IABP removal, the patient encountered rapid clinical deterioration and the emergence of low cardiac output (Table 2). Indeed, the hemodynamic parameters demonstrated a sudden notable elevation in pulmonary pressures and a decline in the cardiac index (CI) from 1.8 to 1.1 L/min/m^2^ (Table 2). Echocardiography revealed massive MR due to a coaptation defect of the leaflets (Figure 1, Supplementary Materials Videos S1 and S2).

Upon escalating the dosage of Sodium Nitroprusside to 1.43 μg/kg/min and Adrenaline to 0.07 ug/kg/min, echocardiography promptly revealed a significant amelioration of the MR grade, with an improvement in the patient’s clinical condition and hemodynamic profile (Table 2). However, the patient remained unstable, with a reiteration of progressive worsening of MR, despite intravenous therapy. Her case was discussed in a multidisciplinary HT and also involved the palliative care physician. Subsequently, a compassionate and emergent MitraClip procedure was scheduled.

The patient underwent percutaneous MitraClip implantation with the placement of two clips while receiving pharmacological inotropic support (Supplementary Materials Videos S3–S6, Table 1). Following the procedure, the inotropic support was gradually tapered, without hemodynamic destabilization. On hospital day 14, echocardiography demonstrated an improvement in LV systolic function with two MitralClips in place and mild residual MR (Supplementary Materials Video S7, Table 2). Medical therapy for HF was started with the introduction of Valsartan (titrated up 40 mg od), Bisoprolol (titrated up to 2.5 mg od), Dapagliflozin (10 mg od), and Eplerenone (titrated up to 50 mg od). The patient was discharged to a rehabilitation center on hospital day 28.

In May 2023, she was admitted for worsening HF in another hospital. Afterwards, due to her unstable condition, she was followed in our outpatient care facility with a monthly infusion of diuretic therapy and intermittent infusion of Levosimendan.

**Figure 2:**
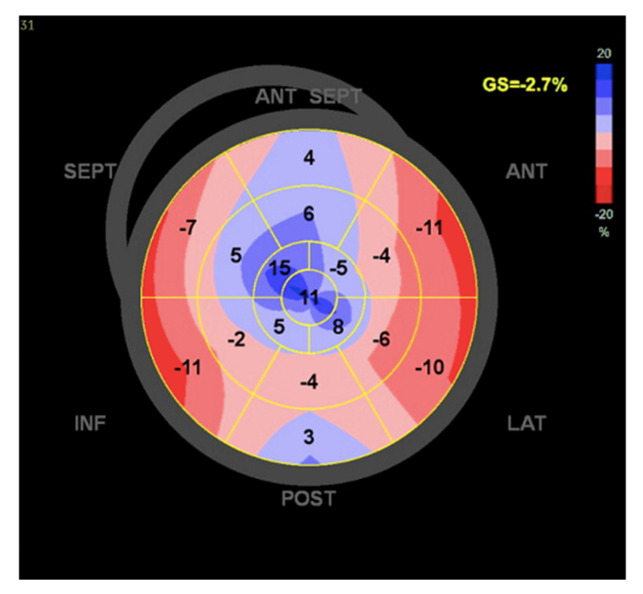
Left ventricle global longitudinal strain.

At the one-year follow-up, the patient was still alive with an LVEF of 15%, a severely depressed LV global longitudinal strain (GLS) (Figure 2), and moderate–severe residual MR (Supplementary Materials Video S8). However, the patient eventually died 15 months following the TEER procedure due to complications of HF.

## 3. Discussion

In this clinical case, cardiogenic shock was treated with a successful MitraClip implantation, which allowed for the stabilization and subsequent discharge of the patient.

Upon admission, the patient presented with a subacute myocardial infarction complicated by hemodynamic instability, which was initially managed with an IABP and pharmacological inotropic support. In this setting, although trials investigating the use of IABP have shown disappointing results, real-world data suggest a possible benefit [7]. However, the clinical scenario was characterized by severe vascular complications, and the IABP was removed. Considering the critical condition of the patient and the encountered severe peripheral arteriopathy, the case was discussed within the HT, and she was excluded from advanced therapies.

Throughout the entire stay in the ICU, severe MR impeded weaning from therapy with inotropes and vasodilators. Through a palliative care physician consultation, the HT decided to proceed with a compassionate and emergent MitraClip implantation. Thus, a multidisciplinary approach was crucial, especially given the increasing complexity of the patient and available treatment options. This clinical case highlights the lack of therapeutic options in patients with cardiogenic shock and advanced heart failure and the potential role of MitraClip implantation in this scenario.

## 4. MitraClip Procedure in Cardiogenic Shock

CS accompanied by valvular heart disease represents a particularly challenging patient population with limited treatment options. Moderate-to-severe MR is observed in 5 to 10% of patients with CS, and its presence is associated with poor outcomes [8]. Given the high surgical risk in these patients, mitral transcatheter edge-to-edge repair (M-TEER) with the MitraClip device may offer a potential treatment option. The ongoing Transcatheter Mitral Valve Repair for Inotrope Dependent Cardiogenic Shock (CAPITAL MINOS) randomized trial [9] is assessing the effectiveness of the MitraClip procedure in this setting, and the results are enthusiastically awaited.

The MitraClip procedure offers advantages for patients with CS and moderate-to-severe MR by providing a prompt and substantial enhancement in forward stroke volume and cardiac output, as well as promoting favorable alterations in LV loading conditions, manifested by a decrease in LV end-diastolic pressure and LV end-diastolic volume [10]. These benefits are particularly appealing given that patients undergoing this procedure avoid the hemodynamic stresses associated with thoracotomy, cardioplegic arrest, and cardiopulmonary bypass.

Awaiting randomized clinical trials, the role of the MitraClip procedure as a salvage therapy in this setting has been documented in observational studies and case series, as reported in Table 3 [11,12,13,14,15,16,17].

A recent study has demonstrated that percutaneous mitral valve repair is safe and effective for patients in CS who are on inotropes or temporary mechanical circulatory support (tMCS) and were excluded from the COAPT trial. The procedure resulted in improved hemodynamic status, evidenced by reduced left atrial pressure and enhanced cardiac output, as well as favorable downward titration of inotropic support [11].

The IRREMI-Registry-based study, which assessed outcomes for patients who developed acute MR following acute myocardial infarction and received M-TEER with the MitraClip device, found no significant difference in outcomes between patients with CS and those who were stable [12]. These results suggest that CS may not be a significant factor in precluding interventional therapy aimed at correcting mitral regurgitation and that MitraClip could be a safe and effective alternative to surgical intervention in clinically unstable patients.

A recent systematic review and meta-analysis collected data from 4060 patients across seven case series and five observational studies on TEER mitral repair in patients with CS [18]. The analysis revealed that the procedure is effective in 88% of cases (post-procedural mitral severity less than 2+), with a 1-year mortality rate of 36%, in-hospital mortality of 11%, and 30-day mortality of 15%. Although the meta-analysis focused solely on mortality in CS patients who underwent TEER, a patient-level multicenter analysis evaluated the effect of MR reduction on survival by comparing outcomes between patients with device success (88.7%) and those without [19]. Their findings indicated that patients who achieved MR reduction had significantly lower in-hospital mortality (Hazard Ratio [HR]: 0.36), as well as decreased mortality at 90 days (HR: 0.36) and 1 year (HR: 0.46) [19].

Consistently, Tang et al. found that TEER repair with MitraClip in patients with MR and CS was associated with lower in-hospital mortality (odds ratio [OR] 0.71) and improved 1-year survival (HR, 0.78) compared to those who did not undergo the MitraClip procedure, with the survival benefit primarily due to mortality events in the non-MitraClip group during the index hospitalization. The authors suggest that one possible explanation is that by reducing MR and improving forward flow, the MitraClip may provide sufficient stabilization to facilitate successful hospital discharge. Moreover, when evaluating the composite endpoint of death, LVAD implantation, or heart transplant, MitraClip use was similarly associated with a survival advantage [20]. In a landmark analysis, after excluding patients who died during the index hospitalization, those who received MitraClip and were discharged alive showed comparable mid-term survival to those who did not undergo the procedure [20]. However, as in the case of our patient, the use of MitraClip as palliative care allows for discharge to home for patients who would otherwise die in the hospital. Data from Tang et al. show that among discharged patients (therefore excluding in-hospital deaths), the MitraClip does not provide a survival benefit but does facilitate discharge [20].

Encouraging data also emerged from a registry-based analysis, which involved a large cohort of 3797 patients with CS who underwent the MitraClip procedure [21]. The study compared outcomes between the device success group (3249 patients, 85.6%) and the device failure group (548 patients, 14,4%). In-hospital mortality was significantly higher in the device failure group (16.4% vs. 9.1%; *p* < 0.001), and successful device deployment was associated with an absolute risk reduction in 1-year mortality of 21%, with a number needed to treat (NNT) of 4.8. However, the 1-year mortality rate for patients with a successful procedure remained high at 38.1% [21]. Nevertheless, concerns have been raised regarding population selection in the analysis, as CS was present in only 804 patients (21.2%), and the majority of the patients qualified solely based on treatment with inotropic support [22].

In summary, MitraClip M-TEER appears to be a promising therapeutic option for patients with mitral regurgitation and cardiogenic shock, a group characterized by extremely high mortality, limited treatment options, and prohibitive surgical risk. The MitraClip procedure has shown effectiveness in over 85% of cases, with low rates of peri-procedural complications. Additionally, it may serve as a salvage therapy to reduce dependence on vasopressors and inotropes.

## 5. MitraClip Procedure in Advanced Heart Failure

Patients with AdHF are estimated to represent 1–10% of the overall HF population, with their prevalence increasing due to improved survival rates [23]. MR plays a crucial role in the progression to end-stage HF, with approximately 25% of AdHF patients experiencing hemodynamically significant MR, ranging from moderate to severe [24]. Data from the HELP-HF registry, which included 1079 HF patients with at least one “I NEED HELP” marker for AdHF, revealed that severe MR was present in 19.2% of cases and was independently associated with an elevated risk of cardiovascular death and recurrent HF hospitalizations [25]. Additionally, the patients with severe MR were more likely to be hospitalized and require intravenous loop diuretics and inotropes/vasopressors [25].

AdHF represents a stage of the disease characterized by high mortality, and therapeutic options in this setting are limited. Particularly, patients with NYHA Class IV experience persistent, refractory symptoms that do not respond adequately to optimal medical therapy, and they are often underrepresented in clinical trials. HTx represents the best option for these patients, but the number of transplants seems to have reached a plateau in the last few years. Furthermore, the population with AdHF is increasing due to HF therapy and is also aging, which often leads to the exclusion of these patients from the option of transplantation due to age or comorbidities. For these reasons, alternative options are emerging.

In advanced stages of HF, some data from the literature suggest the MitraClip procedure as a valid and safe therapeutic option for moderate to severe MR, despite the absence of dedicated clinical trials. Firstly, Franzen et al. analyzed 50 patients with end-stage HF (defined as LVEF ≤25% and NYHA class III/IV) and demonstrated that the MitraClip procedure effectively reduced functional MR at 6 months, with 87% of patients achieving MR ≤2+. Additionally, at 6 months, patients showed significant clinical improvement, with a marked increase in six-minute walk distance and 72% of patients reaching NYHA functional class I or II [26]. A few years later, Berardini et al. evaluated 75 advanced refractory chronic HF patients with secondary MR grade ≥3+ treated with MitraClip implantation. All patients were in NYHA class III/IV before the procedure, 26 patients (35%) were dependent from iv diuretics, and 29 patients (39%) needed iv inotrope infusion. In this study, MitraClip implantation demonstrated improved symptoms, reduced re-hospitalization rates, and lower pro-BNP levels. At 6 months, four patients died (5%), 80% of patients had MR ≤ 2+, and 75% were in New York Heart Association class ≤ II [27]. Subsequently, data from the EXPAND study showed that MitraClip implantation in patients with severe MR (including also primary MR) and NYHA Class IV was found to be safe and effective in treating MR, significantly improving QoL and long-term clinical outcomes. NYHA Class IV subjects showed a significant improvement in MR grade to None/Trace (0) or Mild (1+) in 90.7% of subjects at 30 days and 92.9% of subjects at 1 year [28]. Furthremore, recent subgroup analysis from the COAPT trial demonstrated improved outcomes following MitraClip implantation across all NYHA functional classes (including NYHA IV) [29].

The role of MitraClip in AdHF patients as a bridge to candidacy to HTx has also been evaluated, with initial case reports highlighting its potential in this setting [30,31]. A case series by Crimi et colleagues highlighted its potential role in end-stage HF patients who were initially ineligible or at high risk for HTx due to elevated pulmonary vascular resistance and an unsatisfactory response to vasodilator therapy [32]. In all three cases, the MitraClip procedure led to a sustained reduction in PVR, ultimately allowing these patients to become eligible for HTx [32]. A retrospective study by Geis et al., involving 9 end-stage HF patients listed for HTx with moderate to severe or severe functional MR, demonstrated that the MitraClip procedure results in favorable hemodynamic effects. Specifically, it significantly reduced MR (from grade 3.0 to 1.5), decreased left atrial diameter (from 51 mm to 49 mm), lowered pulmonary artery pressures (sPAP from 50 mmHg to 45 mmHg; mPAP from 34 mmHg to 30 mmHg), and improved mixed-venous oxygen saturation (from 57% to 55%) [33].

Recently, Godino et al. evaluated 119 AdHF patients from the international, multicenter MitraBridge registry. These AdHF patients, who had moderate-to-severe or severe secondary MR, were treated with MitraClip as a bridge strategy under one of the following conditions: those active on the HTx list (bridge to transplant), those suitable for HTx but awaiting a clinical decision (bridge to decision), and those not yet suitable for HTx due to potentially reversible relative contraindications (bridge to candidacy). After one year of follow-up, the MitraClip procedure was demonstrated to be an effective and safe treatment, with two-thirds of patients remaining free from adverse events [34]. These findings were further corroborated by Munafò et al., who conducted a 2-year follow-up of the MitraBridge Registry involving 153 AdHF patients treated with MitraClip. After a median follow-up of 26 months, elective HTx was successfully performed in 30 patients (21%), while 19 patients (13.5%) either maintained or regained eligibility for transplantation. Notably, 22.5% of patients showed such significant clinical improvement that they no longer required a HTx [35].

In conclusion, while the data presented are promising, they remain limited. Since the publication of the COAPT trial, the use of TEER has expanded significantly, even in centers that do not offer advanced therapies [36]. Notably, in patients with advanced HFrEF and severe MR, there is a potential overlap in eligibility for TEER procedures and durable LVAD support, as highlighted in a recent editorial by Noly and colleagues [37].

In this context, patients with refractory HF symptoms and significant MR should be thoroughly evaluated by a multidisciplinary Heart Team, including AdHF specialists [38]. This approach is essential to avoid futile interventions and to determine the optimal timing for advanced HF therapies when indicated. This situation underscores the need for new evidence to guide the selection of the most appropriate treatment option for each patient.

**Table 3:** Overview of selected studies evaluating outcomes of MitraClip implantation in patients with cardiogenic shock and advanced heart failure.

Study	Sample Size	Baseline Population Characteristics	Post Procedural MR <3+	Hospital Stay Length Mean (days)	In-Hospital Mortality n (%)	30-Day Follow- Up	6-Month Follow-Up	1-Year Follow-Up	2-Year Follow-Up
Cheng et al., 2019 [11]	29	CS (defined as at least 1 inotrope and/or required tMCS) with MR ≥ 3+ Mean LVEF (% ± SD) 27.3 ± 16.6	N/A	N/A	5 (17.2)	N/A	Mortality 24.4%	N/A	N/A
Estvez-Loureiro et al., 2021 (IREMMI Registry) [12]	50	Acute MR and CS (according to the CS definition of the Society for Cardiovascular Angiography and Intervention Stage C-E) after AMI Mean LVEF (% ± SD) 34 ± 12 Euroscore II (% ± SD) 21 ± 18	45 (90)	N/A	N/A	Mortality 10%	After median follow-up of 7 months, the combined event mortality/re-hospitalization was 28%	N/A	N/A
Flint et al., 2019 [13]	12	CS (defined as dependence on IV inotrope, IV afterload reduction and/or tMCS immediately preceding MitraClip procedure) with MR Mean LVEF (% ± SD) 46 ± 12 STS score (% ± SD) 33.4 ± 22.3	12 (100)	N/A	1 (8)	Mortality 16.7%	N/A	Mortality 42%	N/A
Pleger et al., 2013 [14]	6	MR in critically ill patients (defined as a patient who could not be weaned from inotropes or from a ventilator, or who was not stable enough to leave the ICU after at least 2 weeks of intensive care treatment) LVEF (%) 20–30 STS score (%) 8–56	6 (100)	N/A	0 (0)	N/A	N/A	Mortality 50%	N/A
Garcia et al., 2020 [15]	11	Severe MR in CS (defined as SBP < 90 mmHg for ≥1 h not responsive to fluid administration alone, thought to be secondary to cardiac dysfunction, and associated with signs of hypoperfusion or CI ≤ 2.2 L/min/mm^2^ and PCWP > 18 mm Hg) LVEF (%) 50 STS score (%) 15	8 (72.7)	N/A	3 (27.3)	Mortality 27.3% MR ≤ 2+ 72.7%	N/A	Mortality 66%	N/A
Chan et al., 2019 [16]	27	Severe MR and refractory CS (defined as the inability to wean inotropic support with or without concomitant IABP or remained ventilator-dependent secondary to pulmonary edema, after at least 7 days of medical optimization) Mean LVEF (% ± SD) 33.5 ± 13.8 STS score (%) 18.5 Euroscore II (%) 27.2	25 (93)	63	8 (30)	Mortality 55.6%	N/A	N/A	N/A
Kovach et al., 2021 [17]	8	Urgent/emergent TEER in CS requiring inotropes/vasopressors or temporary MCS Mean LVEF (%) 46	7 (85)	N/A	4 (50)	Mortality 50%	N/A	N/A	N/A
Jung et al., 2021 [19]	141	MR ≥ 3+ in CS (defined as SCAI stage B to E or requiring inotrope, ventilator, or MCS support) Mean LVEF (% ± SD) 33.8 ± 14.0 STS score (% ± SD) 16.1 ± 16.6	125 (88.6)	10 (6–20)	22 (15.6)	N/A	N/A	Mortality 42.6%	N/A
Tang et al., 2021 [20]	596	MR in patients with CS (International Classification of Diseases, Ninth Edition, [ICD-9] 785.51, International Classification of Diseases, Tenth Revision [ICD-10] R57.0)	N/A	16	148 (24.8)	N/A	N/A	Mortality 56%	N/A
Simard et al., 2022 [21]	3797	MR and CS (defined as the presence of at least 1 of: CS, and/or inotrope use before the procedure, and/or mechanical circulatory support) Mean LVEF (% ± SD) 41.1 ± 17.5 STS score (% ± SD) 14.9 ± 15.3	3249 (85.6)	12.2 ± 14.5	286 (8.8)	Mortality 12.6%	N/A	Mortality 34.6%	N/A
Franzen et al., 2011 [26]	50	End-stage HF (defined as NYHA III/IV and LVEF ≤ 25% despite OMT) with MR ≥ 3+. Mean LVEF (% ± SD) 19 ± 5 EuroSCORE (% ± SD) 34 ± 21	46 (92)	N/A	0 (0)	Mortality 6%	Mortality 18.8% MR ≤ 2+ 87% NYHA I/II 72%	N/A	N/A
Berardini et al., 2016 [27]	75	Advanced refractory chronic HF (defined as severe HF symptoms despite OMT) and MR ≥ 3+ Mean LVEF (% ± SD) 30 ± 9 Euroscore (% ± SD) 23 ± 18 35% of patients were dependent on iv diuretics and 39% needed iv inotropes infusion	63 (84)	N/A	(1.3)	N/A	Mortality 5% MR ≤ 2+ 80% NYHA ≤ II 75%	N/A	N/A
Shuvy et al., 2023 (EXPAND STUDY) [28]	118	NYHA IV with MR (including also primary MR) Mean LVEF (%) 46.79 Euroscore II (% ± SD) 11.15	MR 0/1+ 90.7%	N/A	(0.8)	Mortality 7.7% MR 0/1+ 90.7%	N/A	Mortality 32.4% HFH 29.2% MR 0/1+ 92.9% NYHA I/II 72.6%	N/A
Godino et al., 2020 (MITRABRIDGE Registry) [34]	119	AdHF patients (defined as NYHA III/IV and/or LVEF ≤ 30%) with secondary MR ≥ 3+ treated with MitraClip implantation as BTT (26%)/BTD (45.5%)/BTC (28.5%) Mean LVEF (%) 26 96.5% of patients were in INTERMACS profile ≥ 3 Euroscore II (%) 3.5	N/A	N/A	0 (0)	N/A	Rehospitalizations for HF 4%	Mortality 4.5% Rehospitalizations for HF 30% Urgent HTxs 6% LVAD 5%	N/A
Munafò et al., 2023 (MITRABRIDGE Registry) [35]	153	AdHF patients (defined as NYHA III/IV and/or LVEF ≤ 30%) with secondary MR ≥ 3+ treated with MitraClip implantation as BTT (24.8%)/BTD (52.3%)/BTC (22.8%) Mean LVEF (% ± SD) 26.9 ± 7.7 83.5% of patients were in INTERMACS profile ≥ 3	N/A	N/A	0 (0)	N/A	N/A	N/	Mortality 6.5% First HF rehospitalization 45% LVAD 11.9% (urgent 7%) HTx 21% (urgent HTx 8%) Recovery 22.5%

[AMI, acute myocardial infarction; BP, blood pressure; BTC, bridge to candidacy; BTD, bridge to decision; BTT, bridge to transplant; CI, cardiac index; CS, cardiogenic shock; Htx, heart transplant; ICU, intensive care unit; IV, intravenous; LVEF, left ventricle ejection fraction; LVAD, left ventricular assist device; MCS mechanical circulatory support; MR, mitral regurgitation; PCWP, pulmonary capillary wedge pressure, TEER, transcatheter edge-to-edge repair; tMCS temporary mechanical circulatory support].

## 6. Future Perspectives and Unmet Needs

At the time of this review, robust data from randomized controlled trials testing TEER in AdHF and CS are still lacking. The ongoing CAPITAL MINOS and MITRADVANCE trials are expected to provide valuable insights. Particularly, the MITRADVANCE trial is the first study to compare MitraClip with optimal medical therapy (OMT) in patients with AdHF, and it is expected to provide new data on the efficacy of MitraClip in this population.

A critical evidence gap in this context is the absence of validated prognostic parameters, including clinical, imaging, and hemodynamic variables that can reliably identify the patient profiles most likely to benefit from TEER in this high-risk population. Further research is essential to standardize predictors of TEER efficacy and minimize the risk of futile interventions.

## 7. Conclusions

MitraClip implantation is recognized as a therapeutic option in chronic HF, and promising data in the literature support its use in cardiogenic shock and AdHF. In the absence of randomized studies, it is always recommended to discuss these patients within an HT to ensure the best treatment selection, especially when undecided between LVAD and TEER. In cases where advanced therapeutic options are unavailable, as with our patient, MitraClip implantation proved to be a viable strategy, allowing for discharge at home and, thus, a longer life expectancy.

## Figures and Tables

**Table 1:** Admission laboratory values.

Laboratory Tests	Results	Normal Value
Hemoglobin (g/L)	95	120–160
Creatine kinase (U/L)	2200	34–145
Highly sensitive troponin I (hs-TnI) (ng/L)	70	<34
Creatinine (mg/dL)	0.9	0.3–1.1
BNP (pg/mL)	540	0–100

[BNP = B-Type Natriuretic Peptide]

**Table 2:** Therapies, echocardiographic values, and hemodynamic parameters during the recovery period.

	10 January 2023 (Admission)	13 January 2023	18 January 2023 (IABP Removal)	19 January 2023 2.00 pm	19 January 2023 4.00 pm	21 January 2023 (MitraClip Procedure)	24 January 2023	06 February 2023 (Discharge)
**Inotropic/IABP support**	IABP 100% inflation	IABP 80% inflation, ADN 0.05, NTP 0.16	ADN 0.05, NTP 0.16	ADN 0.05, NTP 0.16	ADN 0.07, NTP 1.43	ADN 0.05, NTP 1.43	ADN 0.025	
**GMDT**							MRA	ACEi, BB, MRA, SGLT2i
**EDVi (BSA 1.65) (mL/m** **^2^)**	106	105	108	115	113		99	96
**LVEF (%)**	25	22	23	20	23		28	35
**DD**	3	2	3	nv	3		nv	nv
**MR (+)**	2+/3+	2+	2+	3+/4+ (Supplementary Materials Videos S1 and S2)	2+/3+	(Supplementary Materials Videos S3–S6)	1+ (Supplementary Materials Video S7)	1+
**LAVi (mL/m^2^)**	52	47	49	54	52		42	39
**TAPSE (mm)**	16	18	17	16	17		18	19
**sPAP (mmHg)**	48	38	nv	57	36		35	32
**SBP (mmHg)**	92	100	97	85	92		95	
**CI (L/min/m^2^)**	1.9	2.1	1.8	1.1	1.9		2.5	
**PAWP (mmHg)**	24	18	18	28	19		18	
**mPAP (mmHg)**	40	17	25	44	16		25	
**Lac (mmol/L)**	2.1	1.1	1.4	2.3	1.5		1.1	
**SvO2 (%)**	55	57	58	50	58		66	

[ACEi = ACE inhibitors; ADN = Adrenaline (μg/kg/min); BB = beta blockers; BSA = body surface area; CI = cardiac index; DD = diastolic dysfunction; EDVi = end-diastolic volume index; GMDT = guideline-directed medical therapy; Lac = lactate; LAVi = left atrial volume index; LVEF = left ventricular ejection fraction; mPAP = mean pulmonary artery pressure; MRA = mineralocorticoid receptor antagonist; MR = mitral regurgitation (+1 mild, +2 moderate, +3 severe, +4 massive); NTP = Sodium Nitroprusside (μg/kg/min); PAWP = wedge pressure; SGLT2i = SGLT2 inhibitors; SBP = systolic blood pressure; sPAP = systolic pulmonary artery pressure; SvO2 = venous oxygen saturation].

## Data Availability

Not applicable.

## Supplementary Materials

The following supporting information can be downloaded at: https://www.mdpi.com/article/10.3390/jcm14031011/s1, Video S1: MR after IABP removal, apical 4-chamber; Video S2: Mitral regurgitation after IABP removal, apical 2-chamber; Video S3: MitraClip procedure part 1; Video S4: MitraClip procedure part 2; Video S5: MitraClip procedure part 3; Video S6: MitraClip procedure part 4; Video S7: MR after MitraClip procedure, apical 4-chamber; Video S8: MR after 1-year follow up.
